# Alexithymia and the labeling of facial emotions: response slowing and increased motor and somatosensory processing

**DOI:** 10.1186/1471-2202-15-40

**Published:** 2014-03-14

**Authors:** Klas Ihme, Julia Sacher, Vladimir Lichev, Nicole Rosenberg, Harald Kugel, Michael Rufer, Hans-Jörgen Grabe, André Pampel, Jöran Lepsien, Anette Kersting, Arno Villringer, Thomas Suslow

**Affiliations:** 1 Department of Psychosomatic Medicine and Psychotherapy, University of Leipzig, Semmelweisstrasse 10, 04103 Leipzig, Germany; 2 Department of Neurology, Max-Planck-Institute of Human Cognitive and Brain Sciences, Leipzig, Germany; 3 Clinic of Cognitive Neurology, University of Leipzig, Leipzig, Germany; 4 Department of Clinical Radiology, University of Münster, Münster, Germany; 5 Department of Psychiatry and Psychotherapy, University Hospital Zurich, Zurich, Switzerland; 6 Department of Psychiatry, University of Greifswald, Greifswald, Germany; 7 HELIOS Hospital, Stralsund, Germany; 8 Nuclear Magnetic Resonance Unit, Max-Planck-Institute of Human Cognitive and Brain Sciences, Leipzig, Germany; 9 Department of Psychiatry, University of Münster, Münster, Germany

**Keywords:** Alexithymia, Supplementary motor area, Somatosensory cortex, Facial emotion, Labeling, Toronto structured interview for Alexithymia

## Abstract

**Background:**

Alexithymia is a personality trait that is characterized by difficulties in identifying and describing feelings. Previous studies have shown that alexithymia is related to problems in recognizing others’ emotional facial expressions when these are presented with temporal constraints. These problems can be less severe when the expressions are visible for a relatively long time. Because the neural correlates of these recognition deficits are still relatively unexplored, we investigated the labeling of facial emotions and brain responses to facial emotions as a function of alexithymia.

**Results:**

Forty-eight healthy participants had to label the emotional expression (angry, fearful, happy, or neutral) of faces presented for 1 or 3 seconds in a forced-choice format while undergoing functional magnetic resonance imaging. The participants’ level of alexithymia was assessed using self-report and interview. In light of the previous findings, we focused our analysis on the alexithymia component of difficulties in describing feelings. Difficulties describing feelings, as assessed by the interview, were associated with increased reaction times for negative (i.e., angry and fearful) faces, but not with labeling accuracy. Moreover, individuals with higher alexithymia showed increased brain activation in the somatosensory cortex and supplementary motor area (SMA) in response to angry and fearful faces. These cortical areas are known to be involved in the simulation of the bodily (motor and somatosensory) components of facial emotions.

**Conclusion:**

The present data indicate that alexithymic individuals may use information related to bodily actions rather than affective states to understand the facial expressions of other persons.

## Background

Understanding the emotional expression of another person is thought to require mimicry or simulation of others’ facial expressions [1,2]. Thus, it is likely that neural assemblies exist that are active both when a person is experiencing and expressing an emotion and when the same person is seeing and interpreting the facial emotions of somebody else [3,4]. Recent evidence indicates that interpreting facial expressions is a multi-faceted endeavor that requires recruiting a multitude of cortical and subcortical circuits, such as the visual system (e.g., occipital gyrus, fusiform gyrus [FFG]), to process the visual information of the face, the motor system for the (covert) physical simulation of the facial movement (supplementary motor area [SMA] or premotor cortex), somatosensory areas for proprioceptive feedback (primary somatosensory cortex, insula) and limbic or frontal regions for reenacting and feeling the according emotion (striatum, ventromedial pre-frontal cortex [vmPFC], amygdala [AMG]) [3-8].

A personality trait that is related to difficulties in the recognition of emotional facial expression is *alexithymia* (literally translated as “*no words for emotion*”). Alexithymia is characterized by deficits in identifying and describing one’s feelings [9]. Alexithymic features can be assessed using the 20-item self-reported Toronto Alexithymia Scale (TAS-20, [10]) or the Toronto Structured Interview for Alexithymia (TSIA, [11]). Both measures of alexithymia include the subscales Difficulties Describing Feelings (DDF), Difficulties Identifying Feelings and Externally Oriented Thinking (the TSIA additionally includes imaginal processing).

It has been repeatedly shown that alexithymia is associated with a decreased ability to identify the facial expressions of others, especially when these expressions are presented under temporal constraints [12-14]. Interestingly, a recent electromyographic (EMG) study demonstrated that highly alexithymic individuals exhibit less facial mimicry when confronted with emotional faces [15]. This could mean that individuals who are high in alexithymia have difficulties in interpreting the emotions of others because they automatically simulate others’ facial expressions to a lesser degree and therefore lack the capability to fully capture the other person’s feelings.

On the contrary, when the presentation time is increased, most studies did not reveal a relationship between the degree of alexithymia and recognition accuracy for emotional facial expressions (e.g., [12,16,17]). So far, only one study [16] has investigated brain activation related to facial emotion labeling, as assessed with longer presentation times (3.75 s) and as a function of alexithymia. No differences as a function of alexithymia could be found. However, the authors studied only 23 participants in a correlational approach. Yarkoni and Braver instead proposed the use of at least 40 participants for a correlational analysis in neuroimaging research [18]. In addition, alexithymic tendencies were only assessed through self-report, although a multi-method approach is recommended [19-21]. Moreover, behavioral evidence [12] suggests that DDF, as opposed to the TAS-20 total score, is most predictive for facial emotion recognition. Thus, the current study investigated the labeling of facial emotions and brain responses to facial emotions as a function of DDF (as measured with TAS-20 and TSIA) using functional magnetic resonance imaging (fMRI). Because our design includes a relatively long response window after the presentation of the facial stimuli, we hypothesized that DDF would have an adverse effect on response latencies but not recognition accuracy.

## Methods

### Participants

Fifty-two healthy young German native speakers (age range: 18 to 29 yrs) participated in the study. All of them were right-handed and had normal or corrected-to-normal visual acuity. None of the participants had any history of neurological or psychiatric illnesses or contraindications for magnetic resonance imaging. All participants gave written consent to participate and received financial compensation for their participation. The study procedure was approved by the ethics committee of the Medical School of the University of Leipzig and was in accordance with the Declaration of Helsinki. Four participants had to be excluded from data analysis (one participant had a depression score of BDI > 14 at time of scanning, one subject displayed excessive head motions in the magnetic resonance imaging (MRI) scanner (>3 mm translation) and two participants demonstrated erroneous reactions and responded before the intended time window). Thus, 48 participants (23 female, age 24 ± 3 yrs, mean ± SD) entered final analysis.

### Assessment of alexithymia and control variables

Alexithymic tendencies were measured using a questionnaire, the TAS-20 (German version: [22]), and an observer-rated measure, the TSIA (German version: [23]). The complete TSIA was administered by one trained interviewer and rated during the interview according to the manual. Before the study, the interviewer was trained to conduct and score the TSIA by the translators of the German version of the TSIA (coauthors MR and HG). This included becoming familiar with the alexithymia construct, the manual outlining administration and the scoring procedures for the TSIA, as well as discussion of the guidelines, the scoring of the items and the correct use of the prompts and probes. Moreover, test interviews were supervised until the interviewer was secure in the solo administration and scoring of the interview. Our analysis was focused on one subscale, DDF, of the TAS-20 and TSIA. This subscale consists of five items in the TAS-20 and six items in the TSIA, respectively. To control for depressive symptoms, anxiety and affectivity, participants also completed the Beck Depression Inventory (German version: [24]), the State-Trait-Anxiety Inventory (German Version: [25]) and the Positive and Negative Affect Schedule (German Version: [26]) trait version.

### Task and design

The participants’ task was to label the facial emotion of a target face. Facial stimuli were color photographs taken from the Karolinska Directed Emotional Face database [27] depicting four different emotions (happy – HA, angry – AN, fearful – FE, and neutral – NE). Pictures of twenty different individuals (ten females) were shown in each of the four emotional conditions, consisting of 80 trials in total. Each trial lasted for 9 s, initiated by the presentation of a fixation cross in the center of the screen for 800 ms. In the first 40 trials of the experiment, the target was shown for 1 s; in the second half of the experiment, the target presentation time was set to 3 s. After presentation of the target, participants had 7.2 (5.2) s to label the emotions by pressing a button. Participants had one response pad per hand with two buttons each and provided their responses with their index and middle fingers. Each emotion was attributed to one button during the entire experiment counterbalanced across participants. During the response window, participants saw the four options in the order of button attribution, e.g., the label on the left side on the screen matched the most left button (i.e., left middle finger). After pressing a button, the labels vanished and only a gray screen was visible until the next trial started with the presentation of the fixation cross. Participants were instructed to answer as correctly as possible within the given time frame and were aware of the fact that the response window was shorter in the second half of the experiment. Trials were shown in two fixed random sequences with the constraints that no two subsequent trials depict the same person and that no more than two subsequent trials show the same emotion.

### MRI acquisition and preprocessing

Structural and functional MR images were obtained on a 3 T scanner (Magnetom Verio, Siemens, Erlangen, Germany). For each participant, structural images were acquired with a T1-weighted 3D MP-RAGE [28]. Magnetization preparation consisted of a non-selective inversion pulse. The imaging parameters were as follows: TI 650 ms, TR 1300 ms, TE 3.5 ms, flip angle 10°, isotropic spatial resolution of 1 mm^3^, two averages. Blood oxygen level dependent contrast sensitive images were collected using T2*-weighted echo-planar imaging (EPI) sequence [matrix 64^2^; resolution 3 mm × 3 mm × 4 mm; gap 0.8 mm; TR 2 s; TE 30 ms; flip angle 90°; interleaved slice acquisition; 385 images]. The slices were oriented parallel to a line through the posterior and anterior commissures.

MRI data were preprocessed and analyzed using SPM8 (http://www.fil.ion.ucl.ac.uk/spm/). The initial five functional volumes were discarded to allow longitudinal magnetization to reach equilibrium. Functional volumes were slice-time corrected (temporal middle slice as reference), realigned to the first image and corrected for movement-induced image distortions (6-parameter rigid body affine realignment). The structural T1 images were coregistered to the mean functional EPI image (default in SPM). Anatomical images were segmented, including normalization to a standard stereotaxic space using the T1 MNI within SPM8. The normalization parameters were then applied to the functional EPI series. The resulting voxel size for the functional images was 3x3x3 mm^3^. A temporal high-pass filter (128 s) was applied to remove slow signal drifts. For the functional data, spatial smoothing was performed using a three-dimensional Gaussian filter of 6 mm full-width at half-maximum. We chose this rather small smoothing kernel such that the potential activation in subcortical areas involved in facial emotion processing was still detectable and not washed out.

### Data analysis

Labeling accuracy was evaluated by the Grier sensitivity index [29], which considers true and false positives. The resulting values for this sensitivity index range from 0 to 1, with a value of 1 meaning perfect performance and a value of 0.5 referring to chance level. Due to the high accuracy and thus lack of sufficient trials to reliably estimate error responses, incorrect trials were discarded prior to analysis of reaction time and fMRI data. The data were pooled across both presentation time conditions. Originally, we aimed to differentiate between the two temporal conditions (1 and 3 s), similar to the study of Parker et al. [12]. However, the accuracy was at its ceiling (> .9) with little variance, such that we decided to collapse across temporal conditions for analysis of reaction time and fMRI data. The high recognition rates in the current study compared to those of Parker et al. seem to be related to our long response window. The participants in Parker et al.’s study had to respond while the picture was presented (1 or 3 s). Participants had more time to respond in the current study, most likely resulting in higher accuracy. This is in line with the conclusions of a recent review (Grynberg et al. [14]), which was published when the data collection for this study was almost finished. Grynberg and colleagues concluded that alexithymic individuals' difficulties in recognizing facial emotions are most prominent when the pictures are presented for less than 300 ms. To investigate associations between measures of alexithymia and labeling accuracy, as well as RTs, correlational analyses were accomplished using Spearman’s rho. Spearman’s rho was also used to check for associations between the measures of alexithymia and affectivity questionnaires (BDI, STAI, and PANAS). We employed Spearman’s rho for correlational analyses because the RT and TSIA-DDF scores were not normally distributed. All associations were tested against a significance threshold of p = .05 (two-tailed).

The fMRI data were analyzed by modeling the onset and duration of the presentation times of each facial expression and by convolving these regressors with the hemodynamic response function for the different emotions. Incorrect trials were included in the first-level design matrix as nuisance regressor. First level t-contrasts were calculated by contrasting each emotional condition with the neutral one (HA > NE, AN > NE, FE > NE). The contrast images for the first level contrasts were then transferred to the second level models for the main effects (HA > NE, AN > NE and FE > NE) and regression models with TAS-20-DDF and TSIA-DDF as regressors. One second level model was calculated per alexithymia measure (TAS-20-DDF, TSIA-DDF) and experimental condition. For all models, significance was tested at the cluster level against a family-wise-error-corrected significance threshold of p = .05 at an individual voxel threshold of t = 3.5. As advised in the literature [30], we also report the activations that would survive a more lenient threshold (p = .001, k = 10) in the additional material to afford using these data in future meta-analyses.

In a recent paper, Yarkoni and colleagues [31] argued that the reaction times per second increase brain activation because the time required for preparatory processes for motor activation is increased. Thus, for contrasts yielding significant clusters, we checked whether adding the difference in RT between the two the conditions in that contrast (e.g., AN > NE) or the RT for the emotion only (e.g., AN) as nuisance covariates changed the results substantially.

Although an association between behavior and TSIA-DDF was revealed for angry and fearful faces, it was only reflected in significant brain activation related to TSIA-DDF in the contrast AN > NE, but not in FE > NE. For FE > NE, the effects on brain activation may be smaller and could thus not be detected using a whole brain approach. Thus, we additionally tested whether there was an association between TSIA-DDF and brain activation in these clusters in an ROI-based approach using small volume correction for FE > NE. For this, the significant clusters from the model testing for a positive correlation between TSIA-DDF and brain activation for the contrast AN > NE were saved as a mask. These, in turn, were employed as an ROI to check for activations positively correlating with TSIA-DDF in these brain areas.

Finally, an exploratory analysis was conducted to check whether our measures of alexithymia (TAS-20, TAS-20-DDF, TSIA, TSIA-DDF) displayed a relationship with brain activations in ROIs, which, based on the previous literature, are associated with facial emotion processing. To estimate the activation in these ROIs, the eigenvariates of the activation in these ROIs were extracted for the main contrasts (i.e., HA > NE, AN > NE, FE > NE) using SPM8. The activations in these ROIs were then related to the measures of alexithymia by employing Spearman’s rho. We decided to employ the following ROIs: amygdala (AMG), ventro-medial pre-frontal gyrus (vmPFC), fusiform gyrus (FFG) and striatum. The masks for AMG, FFG and striatum were defined using the automated anatomical labeling toolbox [32] as implemented in the WFU Pick Atlas [33] using SPM8. However, this tool did not include a reasonable mask for the vmPFC, so we defined this region as a sphere of 20 mm around the MNI coordinates xyz = [0 50–2]. These coordinates were based on the results of a study by Pessoa et al. on facial emotion processing [34]. We also decided to include the clusters (SMA, right S1) positively correlating with TSIA-DDF in the contrast AN > NE as further ROIs.

## Results

### Alexithymia measures and control variables

The mean scores for the alexithymia subscales were 12.4 ± 4.6 (mean ± standard deviation) for the TAS-20-DDF and 2.9 ± 3.4 for the TSIA-DDF. The TAS-20 total score was 43.0 ± 10.7, and the TSIA total score was 16.9 ± 9.9. Internal consistencies for TAS-20-DDF (Cronbach’s α = .87) and TSIA-DDF (α = .90) were sufficiently high. All measures of alexithymia were significantly correlated with each other (see Table 1). There was no correlation between TAS-20-DDF and depression as assessed by the BDI [35], trait-anxiety as measured by the STAI [36], or positive and negative affect as assessed by the PANAS [37] (all ps > .05). TSIA-DDF was not related to BDI or to STAI and PANAS negative (all ps > .05), but there was a negative correlation between TSIA-DDF and PANAS positive (rho = -.33, p < .05). There was a correlation between STAI and BDI (rho = .49, p < .005).

**Table 1:** Correlations (Spearman’s rho) between measures of alexithymia

	**TAS-20**	**TAS-20-DDF**	**TSIA**	**TSIA-DDF**
TAS-20		.85**	.47*	.57**
TAS-20-DDF			.41*	.55**
TSIA				.81**
TSIA-DDF				

*significant at p < .01 (two-tailed).

**significant at p < .001 (two-tailed).

Note. TAS-20 = 20-Item Toronto Alexithymia Scale, TSIA = Toronto Structured Interview for Alexithymia; DDF: subscale Difficulties Describing Feelings.

### Behavioral data

Labeling accuracy was above 0.9 for all facial emotion conditions (happy [HA]: .99, neutral [NE]; .97; angry [AN]: .96; fearful [FE]: .96), and there was no relationship between TAS-20-DDF or TSIA-DDF and performance as measured using the Grier sensitivity index [29] in any of the emotional conditions (all ps > .1). The fastest reaction times were revealed for happy faces, and the slowest reaction times for fearful faces (HA: .73 s, NE: .96 s, AN: 1.06 s, FE: 1.17 s; F(3,141) = 36.4, p < .01; post-hocs: HA < NE = AN < FE). TAS-20-DDF did not correlate with reaction time (RT) in any condition (all ps > .1). However, there was a positive correlation between TSIA-DDF and RT for angry (rho = .30, p < .05) and fearful faces (rho = .31, p < .05), but no correlation was observed between TSIA-DDF and RT for happy (rho = -.01, p = .47) and neutral faces (rho = .07, p = .32) (see Table 2).

**Table 2:** Correlations (Spearman’s rho) between difficulties describing feelings (as assessed by TAS-20-DDF and TSIA-DDF) and reaction times in the four facial expression conditions

		**Reaction times**			
		**Happy**	**Neutral**	**Angry**	**Fearful**
**TAS-20-DDF**	rho	.06	-.04	.15	.11
	p	34	.41	.15	.22
**TSIA-DDF**	rho	-.01	.07	.30*	.31*
	p	.47	.32	.02	.02

*significant at p < .05 (two-tailed).

Note. TAS-20 = 20-Item Toronto Alexithymia Scale, TSIA = Toronto Structured Interview for Alexithymia; DDF: subscale Difficulties Describing Feelings.

### fMRI data

#### Main effects

Happy versus neutral faces elicited significant brain activation in clusters in the left middle occipital gyrus extending to the middle temporal gyrus, in the left middle orbital gyrus extending to both the superior frontal gyrus and the bilateral anterior cingulate gyrus, and a cluster in the middle frontal gyrus extending to the superior frontal gyrus. In the contrast AN > NE, significant clusters were revealed in the right fusiform gyrus, the right inferior occipital gyrus extending to middle occipital and lingual gyrus, the left fusiform gyrus extending to inferior temporal gyrus and the left middle occipital gyrus. The contrast FE > NE activated the left inferior frontal gyrus, left fusiform gyrus extending to inferior occipital gyrus, left middle temporal gyrus, right inferior occipital gyrus and right cerebellar structures (lobule VIIb and VIIa). An overview of the results is presented in Table 3. The activations for the main contrasts are presented at a more lenient threshold (p = .001, k = 10) in the Additional file 1: Table S1.

**Table 3:** Significant brain activations for all fMRI main contrasts

		**Cluster**		**Peak**					**Localization**	
		k	p_fwe_	x	y	z	Z	p_fwe_	hem.	Region
**HA > NE**	1	361	<.001	-42	-76	31	5.10	<.01	Left	Middle occipital gyrus, middle temporal gyrus
	2	461	<.001	-6	53	2	4.99	.01	Left	Middle orbital gyrus, superior frontal gyrus, superior medial gyrus, anterior cingulate gyrus
	3	54	<.05	-30	26	49	4.43	.16	Left	Middle frontal gyrus, superior frontal gyrus
**AN > NE**	1	98	<.01	42	-49	-14	4.63	.05	Right	Fusiform gyrus
	2	81	<.01	36	-91	4	4.98	.11	Right	Inferior occipital gyrus, middle occipital gyrus, lingual gyrus
	3	121	<.001	-39	-73	-8	4.40	.13	Left	Fusiform gyrus, inferior temporal gyrus
	4	48	<.05	-30	-94	7	3.92	.52	Left	Middle occipital gyrus
**FE > NE**	1	615	<.001	-45	14	22	5.93	<.001	Left	Inferior frontal gyrus, pars triangularis
	2	167	<.001	36	-91	2	5.58	<.001	Right	Inferior occipital gyrus
	3	292	<.001	-36	-73	-8	5.03	<.01	Left	Fusiform gyrus, inferior occipital gyrus
	4	106	<.01	-57	-55	7	4.62	.05	Left	Middle temporal gyrus
	5	102	<.01	15	-79	-35	4.58	.06	Right	Cerebellum, lobule VIIb

Note. Only clusters that are significant on cluster level (p_fwe_ < 0.05) at an individual voxel threshold of t = 3.5 are reported. The region refers to brain areas through which the cluster is spanning. HA > NE = happy versus neutral faces, AN > NE = angry versus neutral faces, FE > NE = fearful versus neutral faces, hem. = hemisphere, x, y and z are in MNI space.

#### Relationships between brain activation and measures of alexithymia

**Figure 1:**
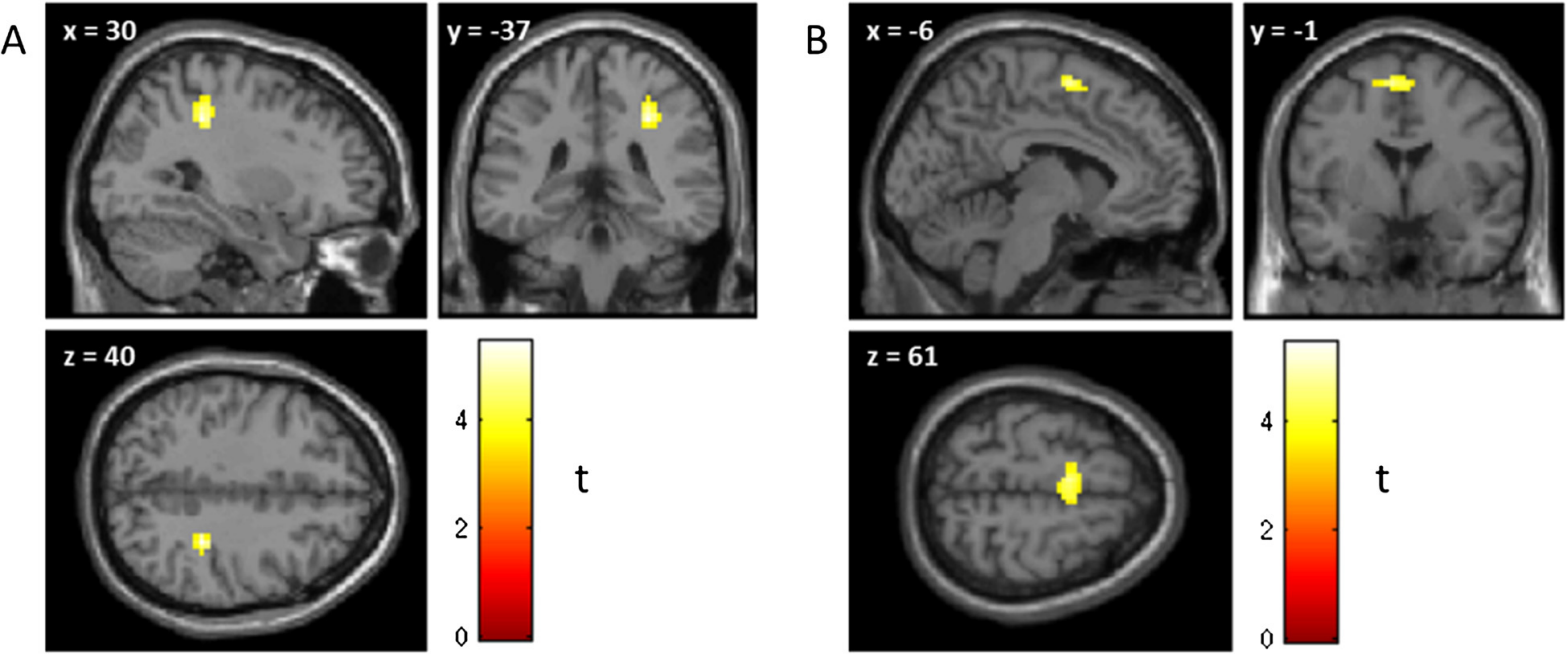
Activation in the right postcentral gyrus (A) and supplementary motor area (B) in response to angry (vs. neutral) faces (AN > NE) positively correlating with TSIA-DDF.

A significant cluster positively correlating with TSIA-DDF in the contrast AN > NE was revealed in the supplementary motor area (SMA) (Montreal Neurological Institute [MNI] coordinates xyz = [-6 -1 61], cluster extent k = 64, p_cluster_ = .013). The peak of this cluster lay in SMA proper, but the activity clearly extended more rostrally to pre-SMA. A second cluster was found in the post-central gyrus (xyz = [30–37 40], p_cluster_ = .039, k = 47) (see Figure 1), which could be attributed to the right primary somatosensory (S1) cortex. No significant clusters were revealed related to TAS-20-DDF or any other contrast for TSIA-DDF. Because TSIA-DDF was negatively correlated with the positive affect, we checked whether entering the PANAS positive score as a nuisance covariate into the model affected the results. We found a small change for the cluster in the SMA (xyz = [-6 -1 61], k = 39, p_cluster_ = .089) and a decrease in cluster size in the somatosensory cortex (xyz = [30–37 40], k = 14, p_cluster_ = .45, p_peak-uncorrected_ < .0001).

Entering the difference between the reaction times for AN and NE as nuisance covariates into our second-level model slightly changed the results (SMA: xyz = [3 2 61], k = 43, p_cluster_ = .05; somatosensory cortex: xyz = [30–37 40], k = 47, p_cluster_ = .038). Similarly, using only the reaction time in the angry condition as a covariate led to small changes in the results (SMA: xyz = [3 2 61], k = 37, p_cluster_ = .08; somatosensory cortex: xyz = [30–37 40], k = 45, p_cluster_ = .046). Thus, our findings are highly likely to mainly reflect differences due to alexithymia (TSIA-DDF) and cannot be attributed to (differences in) the reaction time. The activations for the models related to the measures of alexithymia are presented at a more lenient threshold (p = .001, k = 10) in the Additional file 2: Table S2.

#### Post-hoc analysis of activation in SMA and S1 positively correlating with TSIA-DDF for contrast FE > NE

A post-hoc region of interest (ROI) analysis revealed a significant small-volume-corrected (SVC) peak voxel activation in the SMA (xyz = [-9 -4 61], p_SVC_ = .019) and a marginally significant peak voxel activation in S1 (xyz = [30–37 40], p_SVC_ = .069) positively correlating with TSIA-DDF in the contrast FE > NE. The activation in SMA remained marginally significant when controlling for PANAS positive affect (p_SVC_ = .061) and significant when entering the difference in RT between FE and NE (p_SVC_ = .032), or RT in FE alone (p_SVC_ = .034). Similarly, the significance of the activation in S1 changed only slightly when controlling for PANAS (p_SVC_ = .084), the difference in RT (p_SVC_ = .052) or the reaction time for FE (p_SVC_ = .059).

#### Exploratory analysis of correlations between brain regions relevant for emotion processing and measures of alexithymia

**Figure 2:**
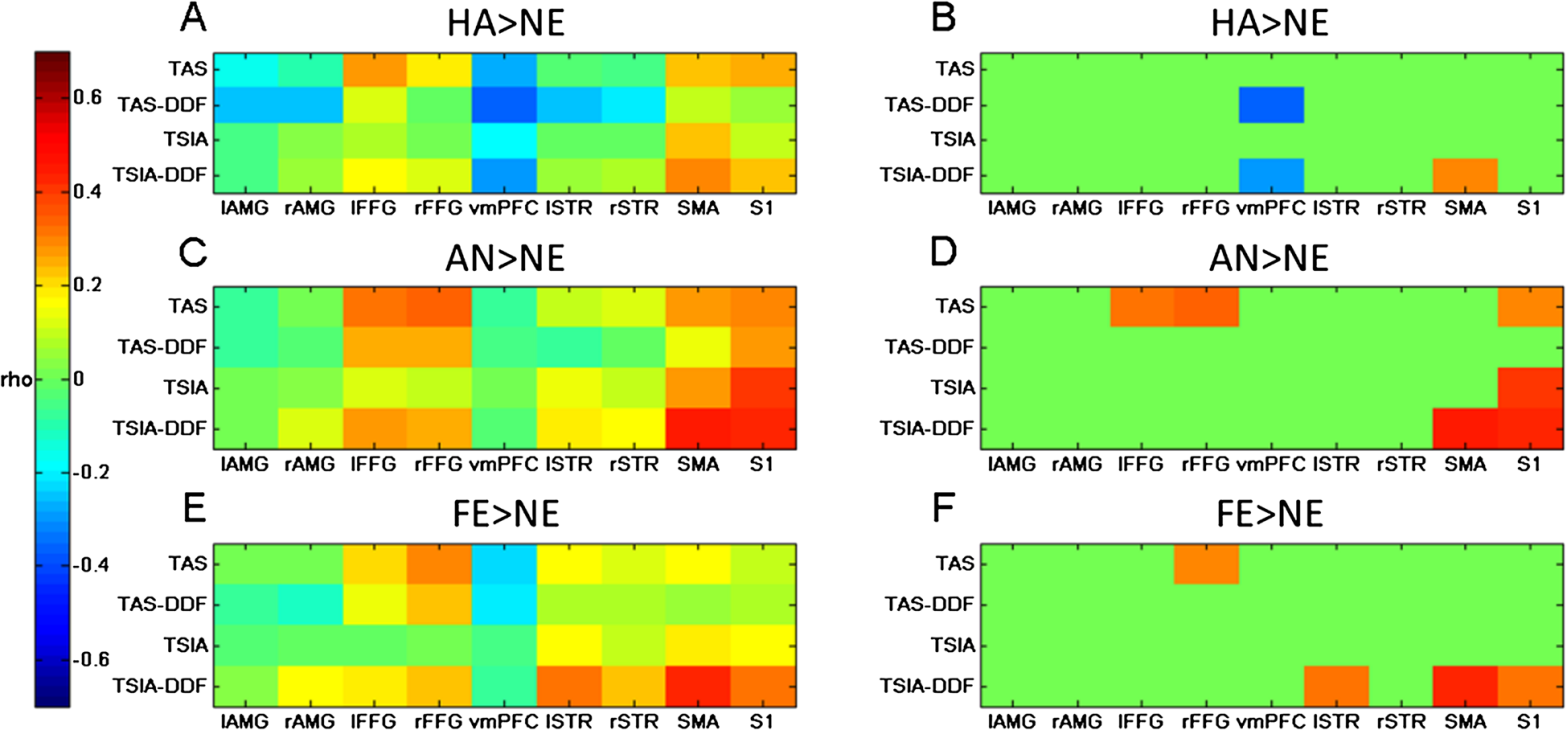
**Relationship (as calculated with Spearman’s rho) between measures of alexithymia and brain activations in regions-of-interest that are relevant for facial emotion processing.** The left column **(A,C,E)** depicts the magnitude of rho coded by color; in the right column **(B,D,F)**, rho is only presented as different from zero if the according p < .05. Each row is related to one contrast: HA > NE (happy > neutral) in **A** and **B**. AN > NE (angry > neutral) in **C** and **D**. FE > NE (fearful > neutral) in **E** and **F**. l = left, r = right; AMG = amygdala, FFG = fusiform gyrus, vmPFC = ventro-medial pre-frontal cortex, STR = striatum, SMA = supplementary motor area, S1 = primary sensory cortex.

The results of our exploratory analysis considering associations between measures of alexithymia and brain activity in the AMG, FFG, vmPFC, striatum, SMA and S1 are displayed in Figure 2. Descriptively, our measures of alexithymia are rather positively related to activation in S1 and SMA and show no or negative correlative trends with AMG and vmPFC. These relationships between alexithymia and FFG as well as striatum depend on the contrast, and no consistent pattern emerges. When thresholding the plot at p = .05 (two-tailed), SMA seems to be strongly associated with TSIA-DDF (all contrasts), while S1 is related to TSIA-DDF (AN > NE, FE > NE), TSIA and TAS-20 (AN > NE) in the conditions with negative emotions. For the contrast HA > NE, the activity in vmPFC is negatively related to TAS-20-DDF and TSIA-DDF. Moreover, FFG activity seems to be positively related to TAS-20 in both negative conditions. For AN > NE, activation in the left striatum seems to be positively related to TSIA-DDF. However, it has to be noted that the correlations between TSIA-DDF and SMA and S1 for AN > NE are likely to be an overestimation of the real correlations in these areas because we extracted a cluster using a mask defined by voxels that positively correlated with TSIA-DDF in that very contrast (cf. [38,39]). Thus, these correlations are only presented here in an exploratory and descriptive fashion.

## Discussion

This study investigated the effects of self-report (TAS-20-DDF) and observer-rated (TSIA-DDF) facets of alexithymia on the labeling and neural processing of facial emotions presented for a rather long time (1 or 3 seconds). Our analysis of the main contrasts revealed significant clusters of brain activation in the fusiform gyrus, inferior and middle occipital gyrus (all conditions), in the middle temporal gyrus (fearful faces), inferior (fearful) and orbital and medial (happy) frontal gyrus as well as the cerebellum. All of these regions have been reported to be implicated in facial emotion processing (e.g.: [7,8,40-42]). Thus, we can assume that our experimental design is suitable for eliciting brain activation related to facial emotion recognition. Considering the specific effects of alexithymia, we found that high TSIA-DDF scores were related to increased reaction times when labeling angry and fearful faces and to increased brain activation in SMA and right S1 during the recognition of these negative faces. A post-hoc exploratory analysis suggests that activity in brain areas that are important in the affective components of facial emotion processing (AMG, vmPFC, striatum) does not show a particular relationship with alexithymia in the current task.

Their increased reaction times indicate that alexithymic individuals were slower in labeling negative emotions. Highly alexithymic individuals appear to need more time to reach a labeling accuracy level similar to subjects with low alexithymia. In contrast to previous studies describing a relationship between accuracy and degree of alexithymia [12,13], we used relatively long stimulus presentation times and response windows and could not reveal interrelationships between alexithymia and recognition accuracy. Thus, it seems that alexithymic individuals have difficulties in recognizing facial expressions, which are reflected in decreased accuracy when presentation times and response windows are short (see also [14]). Prolonging presentation times and response windows could improve recognition accuracy, however, at the cost of increases in response time.

SMA is part of a brain network that is involved in the processing of motor-related information and motor preparation and has been shown to be involved in the production of facial emotions [43]. Moreover, it has been argued that (especially pre-) SMA is involved in the recognition of facial emotions [44] by playing an important role in the motor components of simulation (see also [6]). Additionally, a cluster in S1 was revealed, which seems to reflect somatosensory aspects of facial emotion processing [3,7,45]. According to Adolphs et al. [46], recognizing emotions from facial expressions requires right primary somatosensory areas. The authors argue that recognition of another individual’s emotional state is mediated by internally generated somatosensory representations that simulate how the other individual would feel when displaying a certain facial expression. Taken together, this mediation could mean that highly alexithymic individuals have difficulties in automatically reenacting the negative facial emotion of others when these are presented briefly [15]. When the presentation time is increased, highly alexithymic individuals can reach a similar performance as less alexithymic individuals, which seem to require an increased activation of motor and somatosensory areas. Interestingly, it has been found that highly (as compared to less) alexithymic individuals also show increased activation in motor-related brain areas when interpreting the directed actions of others in a classical mirror-neuron task and show no differences in interpreting these actions [47]. Thus, highly alexithymic individuals may be more inclined to imitate the actions of others via (covert) motor simulation than are non-alexithymics. A recent meta-analysis by van der Velde et al. [48] reported that high levels of alexithymia are related to decreased activity in the SMA when participants are confronted with negative stimuli. However, this meta-analysis included all types of emotional paradigms and tasks (not only facial emotion recognition), so the published results may not necessarily reflect processes related specifically to facial emotion recognition.

There seems to be no particular relationship between activity in the amygdala, vmPFC and ventral striatum and alexithymia in the task studied here. This finding is very interesting because earlier studies on brain function [49-52] and structure [53] reported alterations in highly alexithymic individuals in these regions. In particular, functional studies on automatic processing of emotional faces (affective priming) [49-51] have revealed decreased activations in these brain areas. The lack of involvement in the current task may be the case because the emotional faces were presented for a rather long time in the current study. The amygdala and the ventral striatum, however, are thought to operate in a fast and automatic fashion and may be less relevant when the participants are fully aware of the emotional nature of the faces (e.g., [54,55]), as in the current study. Thus, it seems that alexithymic individuals show less automatic activation in brain regions particularly involved in the affective components of face processing (AMG, ventral striatum, vmPFC), which most likely leads to alterations in the processing of and difficulties in the labeling of briefly presented faces. However, alexithymic individuals seem to be able to simulate the bodily aspects of facial expressions when the presentation times and response windows are long enough, which makes the correct recognition of faces possible in this case.

Our study points to deficits limited to the recognition of negative faces in alexithymia. Neither behavioral nor neurobiological differences were revealed for happy faces. This finding suggests that alexithymics have fewer problems interpreting positive compared to negative facial expressions. A recent review on alexithymia and the processing of emotional facial expressions concluded that the difficulties of alexithymic individuals in processing facial emotions are not specific to certain emotions [14]. The work of Sonnby-Borgström [15] shows that the imitation of facial expressions (measured with facial EMG) in highly alexithymic individuals was only decreased for corrugator activity related to negative emotions, but not for zygomaticus activity related to happy faces. Against this background, alexithymic individuals may display fewer deficits in automatically simulating happy faces compared to neutral ones, which possibly renders the recognition of happy faces easier.

It is important to note that in our study, the objective measure of alexithymia (TSIA), but not the self-report measure (TAS-20), was predictive for recognition performance. Because some alexithymic individuals may not be aware of their own deficits, self-report tests could be less suitable for measuring difficulties in describing feelings compared to objective tests such as the TSIA.

It has been argued that the TAS-20 and the TSIA only measure cognitive aspects of alexithymia and neglect affective parts of the alexithymia construct [56]. A questionnaire that possibly captures these affective components is the Bermond-Vorst-Alexithymia Questionnaire ( [57], but see also [58]). It is possible that additionally applying this measure of alexithymia may have the potential to discover relationships between the brain areas involved in the affective components of emotional face processing. Future studies need to be conducted to determine whether the results of the current study are only related to cognitive alexithymia or whether they generalize to affective alexithymia as well.

## Conclusion

In summary, alexithymic individuals have difficulties in labeling facial expressions of emotion, even when these are presented with little temporal constraints. Such individuals are slowed in their labeling of angry and fearful facial emotions, and they manifest increased activation in the somatosensory and supplementary motor cortex in response to these negative faces. These cortical regions are involved in the simulation of the bodily components of facial emotional expressions. Thus, the present data suggest that alexithymic individuals may recruit cortical processing resources that are involved in the simulation of the bodily components rather than of affective states (angry and fearful) to interpret these facial expressions.

## Abbreviations

AMG: Amygdala; AN: Experimental condition with angry faces; BDI: Beck depression inventory; DDF: Difficulties describing feelings (subscale of TAS-20 and TSIA); EMG: Electromyography; EPI: Echo planar imaging; FE: Experimental condition with fearful faces; FFG: Fusiform gyrus; (f)MRI: (functional) magnetic resonance imaging; HA: Experimental condition with happy faces; MNI: Montreal neurological institute; NE: Experimental condition with neutral faces; PANAS: Positive and Negative affect schedule; ROI: Region of interest; RT: Reaction time; S1: Primary somatosensory cortex; SMA: Supplementary motor area; STAI: State-trait-anxiety inventory; SVC: Small volume corrected; TAS-20: 20-Item Toronto Alexithymia scale; TSIA: Toronto structured interview for Alexithymia; vmPFC: Ventro-medial prefrontal cortex.

## Competing interest

The authors declare that they have no competing interests.

## Authors’ contributions

KI, JS, VL, NR, HK, MR, HG, AP, JL, AK, AV, TS designed the study; MR, HG supervised the alexithymia interviews; KI, VL, NR, TS conducted the psychometric testing of the participants; JS, HK, AP, JL, AV prepared the fMRI sequences; KI, JS, VL, NR, TS, AP run the fMRI experiments; KI, VL, NR, MR, HG, TS analyzed the psychometric data; KI, JS, TS analyzed the fMRI data; KI, JS, VL, NR, HK, MR, HG, AP, JL, AK, AV, TS interpreted the data; KI, JS, AK, TS wrote the manuscript. All authors read and approved the final manuscript.

## Supplementary Material

Additional file 1: Table S1 Brain activation in the three main contrasts at a threshold of t = 3.27, k = 10. Click here for file

Additional file 2: Table S2 Brain activation related to measures of alexithymia in the three contrasts at a threshold of t = 3.27, k = 10. Click here for file
