# Supplementary material for: Alexithymia and the labeling of facial emotions: response slowing and increased motor and somatosensory processing
Source: BMC Neurosci. 2014 Mar 14;15:40. doi: 10.1186/1471-2202-15-40 (PMC4003818; doi:10.1186/1471-2202-15-40)
Supplement: Additional file 1: Table S1 — Brain activation in the three main contrasts at a threshold of t = 3.27, k = 10. [file 1471-2202-15-40-S1.doc]

Additional file 1: Table S1. Brain activation in the three main contrasts at a threshold of t=3.27, k=10.

|  |  |  |  | **cluster** | |  | **peak** | | | | |  | **localization** | |
| --- | --- | --- | --- | --- | --- | --- | --- | --- | --- | --- | --- | --- | --- | --- |
|  |  |  |  | k | p_fwe_ |  | x | y | z | Z | p_fwe_ |  | hem. | region |
|  |  |  |  |  |  |  |  |  |  |  |  |  |  |  |
| **HA>NE** |  | 1 |  | 429 | <.001 |  | -42 | -76 | 31 | 5.10 | <.01 |  | left | middle occipital gyrus, middle temporal gyrus |
|  |  | 2 |  | 571 | <.001 |  | -6 | 53 | 2 | 4.99 | <.05 |  | left | middle orbital gyrus, superior frontal gyrus, superior medial gyrus, anterior cingulate gyrus |
|  |  | 3 |  | 109 | <.01 |  | -30 | 26 | 49 | 4.43 | .16 |  | left | middle frontal gyrus, superior frontal gyrus |
|  |  | 4 |  | 33 | .24 |  | -6 | -58 | 19 | 3.93 | .52 |  | left | precuneus |
|  |  | 5 |  | 14 | .70 |  | -24 | -7 | -20 | 3.93 | .52 |  | left | amygdala |
|  |  | 6 |  | 46 | .11 |  | -9 | -46 | 34 | 3.88 | .58 |  | left | middle cingulate gyrus |
|  |  | 7 |  | 20 | .52 |  | -27 | -34 | -14 | 3.74 | .74 |  | left | parahippocampal gyurs |
|  |  | 8 |  | 17 | .60 |  | -63 | -25 | -14 | 3.66 | .83 |  | left | middle temporal gyrus |
| **AN>NE** |  | 1 |  | 140 | <.01 |  | 42 | -49 | -14 | 4.63 | .05 |  | right | fusiform gyrus |
|  |  | 2 |  | 103 | <.01 |  | 36 | -91 | 4 | 4.98 | .11 |  | right | inferior occipital gyrus, middle occipital gyrus, lingual gyrus |
|  |  | 3 |  | 219 | <.001 |  | -39 | -73 | -8 | 4.40 | .13 |  | left | fusiform gyrus, inferior temporal gyrus |
|  |  | 4 |  | 77 | <.05 |  | -30 | -94 | 7 | 3.92 | .52 |  | left | middle occipital gyrus |
|  |  | 5 |  | 45 | .13 |  | 21 | -10 | -14 | 4.84 | <.05 |  | right | amygdala, hippocampus |
|  |  | 6 |  | 24 | .42 |  | 51 | -37 | 4 | 4.29 | .19 |  | right | middle temporal gyrus |
|  |  | 7 |  | 37 | .20 |  | -39 | 32 | -5 | 4.28 | .18 |  | left | inferior frontal gyrus |
|  |  | 8 |  | 17 | .61 |  | 51 | 35 | 1 | 4.09 | .34 |  | right | inferior frontal gyrus |
|  |  | 9 |  | 37 | .20 |  | -21 | -7 | 17 | 4.03 | .41 |  | left | amygdala |
|  |  | 10 |  | 20 | .52 |  | -3 | -31 | -11 | 3.96 | .48 |  | left | not found in probability map |
|  |  | 11 |  | 62 | .05 |  | -51 | 5 | 28 | 3.89 | .57 |  | left | precentral gyrus, inferior frontal gyrus |
|  |  | 12 |  | 29 | .31 |  | -3 | -13 | 4 | 3.76 | .71 |  | left | thalamus |
|  |  | 13 |  | 13 | .74 |  | -6 | 53 | 31 | 3.47 | .95 |  | left | superior medial gyrus |
| **FE>NE** |  | 1 |  | 721 | <.001 |  | -45 | 14 | 22 | 5.93 | <.001 |  | left | inferior frontal gyrus, pars triangularis |
|  |  | 2 |  | 203 | <.001 |  | 36 | -91 | 2 | 5.58 | <.001 |  | right | inferior occipital gyrus |
|  |  | 3 |  | 350 | <.001 |  | -36 | -73 | -8 | 5.03 | <.01 |  | left | fusiform gyrus, inferior occipital gyrus |
|  |  | 4 |  | 130 | <.01 |  | -57 | -55 | 7 | 4.62 | .05 |  | left | middle temporal gyrus |
|  |  | 5 |  | 145 | <.01 |  | 15 | -79 | -35 | 4.58 | .06 |  | right | cerebellum, lobule VIIb |
|  |  | 6 |  | 53 | .09 |  | -6 | 23 | 49 | 4.29 | .13 |  | left | supplementary motor area |
|  |  | 7 |  | 63 | .06 |  | 48 | -37 | 4 | 4.15 | .27 |  | right | middle temporal gyrus |
|  |  | 8 |  | 20 | .53 |  | 42 | 17 | 25 | 3.98 | .44 |  | right | inferior frontal gyrus |
|  |  | 9 |  | 14 | .71 |  | -6 | -10 | 4 | 3.91 | .52 |  | left | thalamus |
|  |  | 10 |  | 24 | .44 |  | 42 | -49 | 14 | 3.65 | .81 |  | right | inferior temporal gyrus |

Note. The region refers to brain areas through which the cluster is spanning. HA>NE = happy versus neutral faces, AN>NE = angry versus neutral faces, FE>NE = fearful versus neutral faces, hem. = hemisphere, x,y and z are in MNI space.
