# Supplementary material for: Alexithymia and the labeling of facial emotions: response slowing and increased motor and somatosensory processing
Source: BMC Neurosci. 2014 Mar 14;15:40. doi: 10.1186/1471-2202-15-40 (PMC4003818; doi:10.1186/1471-2202-15-40)
Supplement: Additional file 2: Table S2 — Brain activation related to measures of alexithymia in the three contrasts at a threshold of t = 3.27, k = 10. [file 1471-2202-15-40-S2.doc]

Additional file 2: Table S2. Brain activation related to measures of alexithymia in the three contrasts at a threshold of t=3.27, k=10.

|  |  | | **cluster** | | |  | **peak** | |  |  |  |  | **localization** | |
| --- | --- | --- | --- | --- | --- | --- | --- | --- | --- | --- | --- | --- | --- | --- |
|  |  | | k | p_fwe_ | |  | x | y | z | Z | p_fwe_ |  | hem. | region |
|  |  | |  |  | |  |  |  |  |  |  |  |  |  |
| **HA>NE** | TSIA-DDF+ | | 14 | .70 | |  | 30 | -31 | 34 | 3.92 | .52 |  | right | not assigned (close to posterior gyrus) |
|  | TSIA-DDF- | | 16 | .64 | |  | -3 | 53 | 4 | 3.86 | .60 |  | left | middle orbital gyrus |
| **AN>NE** | TSIA-DDF+ | | 61 | .05 | |  | 30 | -37 | 40 | 4.76 | <.05 |  | right | Area 3a, S1 |
|  |  | | 87 | <.01 | |  | -6 | -1 | 61 | 4.20 | .24 |  | left | Area 6, supplementary motor area |
|  |  | | 16 | .64 | |  | 54 | -28 | 7 | 3.78 | .69 |  | right | superior parietal lobule |
|  |  | | 23 | .44 | |  | -18 | -58 | 52 | 3.71 | .76 |  | left | superior temporal gyrus |
|  |  | | 11 | .80 | |  | -21 | -49 | 49 | 3.67 | .81 |  | right | Area 6, precentral gyrus |
|  | TSIA-DDF-: no suprathreshold activation | | | | | | | | | |  |  |  |  |
| **FE>NE** | TSIA-DDF+ | 11 | | | .80 |  | -54 | 11 | 19 | 3.78 | .67 |  | left | Area 44, inferior frontal gyrus |
|  |  | 11 | | | .80 |  | 9 | -4 | 61 | 3.39 | .96 |  | right | Area 6, supplementary motor area |
|  | TSIA-DDF- | 15 | | | .68 |  | -9 | -4 | 25 | 3.62 | .83 |  | left | caudate nucleus |

Note. No suprathreshold clusters were revealed in relation to the subscale DDF of the 20-item Toronto Alexithymia Scale. The region refers to brain areas through which the cluster is spanning. HA>NE = happy versus neutral faces, AN>NE = angry versus neutral faces, FE>NE = fearful versus neutral faces, TSIA = Toronto Structured Interview for Alexithymia. DDF = subscale difficulties describing feelings, + = positive relationship between measure and brain activation, - = negative relationship between measure and brain activation, hem. = hemisphere, x,y and z are in MNI space.
